# Genomic and Phenotypic Characterization of *Campylobacter fetus* subsp. *venerealis* Strains

**DOI:** 10.3390/microorganisms9020340

**Published:** 2021-02-09

**Authors:** Marta F. Silva, Ana L. Pereira, Maria J. Fraqueza, Gonçalo Pereira, Luísa Mateus, Luís Lopes-da-Costa, Elisabete Silva

**Affiliations:** CIISA—Centro de Investigação Interdisciplinar em Sanidade Animal, Faculdade de Medicina Veterinária, Universidade de Lisboa, Av. da Universidade Técnica de Lisboa, 1300-477 Lisboa, Portugal; martasilva@fmv.ulisboa.pt (M.F.S.); anapereira271268@yahoo.com (A.L.P.); mjoaofraqueza@fmv.ulisboa.pt (M.J.F.); goncalopereira@fmv.ulisboa.pt (G.P.); lmateus@fmv.ulisboa.pt (L.M.); lcosta@fmv.ulisboa.pt (L.L.-d.-C.)

**Keywords:** *Campylobacter fetus* subsp. *venerealis*, biovar intermedius, multilocus sequencing typing, virulence potential, antimicrobial susceptibility, genomic characterization

## Abstract

The pathogenesis mechanisms of *Campylobacter fetus* subsp. *venerealis* (*Cfv*), the etiologic agent of Bovine Genital Campylobacteriosis remain elusive. This study evaluated the virulence potential and biovar characteristics of *Cfv* isolates (*n* = 13) by PCR screening of putative virulence-factor (VF) genes, Multilocus Sequence Typing (MLST) analysis, antimicrobial susceptibility to tetracycline, penicillin, enrofloxacin and streptomycin testing and whole-genome sequencing (WGS; *n* = 5), also comparing the latter with 26 other whole-genome sequences of *Cfv* strains. The putative VF genes encoding type IV secretion system of *Cfv* (*virB2*-*virB11*/*virD4*) were absent in 92% of isolates, including isolates from aborted foetuses, evidencing that these VF genes are not essential for *Cfv* pathogenicity. The *parA* gene, used as a *Cfv* diagnostic molecular target, was detected in only 3 of 13 isolates, invalidating its use for diagnosis purposes. Three novel sequence types were identified by MLST. Although no in vitro antimicrobial resistance was detected, WGS identified antimicrobial resistance-related genes, including those encoding the multidrug efflux pumps CmeABC and YkkCD, indicating that their presence is not enough to provide antimicrobial resistance. The SNP and accessory protein families analysis segregated the *Cfv* and *Cfv* biovar intermedius (*Cfvi*) strains into different clusters. In conclusion, this study evidenced virulence potential and biovar characteristics of *Cfv* and *Cfvi*, which are of relevance for the control of Bovine Genital Campylobacteriosis.

## 1. Introduction

*Campylobacter fetus* subsp. *venerealis* (*Cfv*) is the etiological agent of Bovine Genital Campylobacteriosis (BGC), a notifiable venereal disease of cattle responsible for low herd reproductive efficiency and significant economic losses worldwide [1,2]. Infected bulls asymptomatically carry *Cfv* in the preputial and penile mucosa and infect females during natural breeding or through semen, causing embryo loss or early fetal abortion [3]. For that reason, BGC control is based on bull preputial testing and culling of infected bulls, which requires an accurate identification of *Cfv* [3,4].

The Multilocus Sequencing Typing (MLST) identified a clonal structure with lower genetic diversity among *C. fetus* isolates than among other *Campylobacter* species [5]. In fact, *C. fetus* subsp. *fetus* (*Cff*), which colonizes the intestinal tract and occasionally the preputial cavity, causing sporadic abortion in cattle, and *Cfv* have more than 90% genome similarity [6]. This hampers the selection of suitable molecular targets for subspecies identification. The subspecies *Cfv* includes the biovar intermedius, which is differentiated by its ability to produce hydrogen sulfide from L-cysteine [7,8]. However, this is also common to *Cff*, hindering the subspecies differentiation by phenotypic tests. The determinants behind *Cfv* pathogenicity and niche restriction are still unclear. Several putative virulence factor (VF) genes were identified in both subspecies, including those encoding proteins involved in bacterial adhesion, invasion and cytotoxicity, which are common to other *Campylobacter* species, namely, the fibronectin binding protein, the campylobacter invasion antigen (Ciab) and the cytolethal distending toxin (CDT), among others [9]. Nevertheless, comparative genomic analyses revealed the presence of a genomic island almost exclusive of *Cfv* and highly prevalent in this subspecies [9,10,11], which harbors one of the most well studied genes for *Cfv* identification, the *parA* gene [12,13]. This genomic island harbors genes encoding Fic (filamentation induced by cyclic AMP)—domain proteins (*fic* genes) and a bacterial type IV secretion system (T4SS, *virB*-*virD4* genes) [10]. In vitro studies demonstrated that the T4SS contributes to cytotoxicity and invasiveness of *Cfv*, besides being involved in interbacterial DNA transfer by conjugation [10,14]. Additionally, Fic proteins form a toxin–antitoxin network in *Cfv* that may favor its survival under adverse conditions [15]. These findings indicate a role of this genomic island in *Cfv* pathogenicity and/or adaptation to the genital tract [10,15]. A recent study revealed that *C. fetus* strains commonly harbor multiple T4SS encoding regions, which are phylogenetically different and were possibly acquired from different *Campylobacter* species [16]. Nevertheless, some T4SS encoding regions lack several *virB* genes and their function is still unclear.

Bulls may be treated with antibiotics, namely penicillin and streptomycin, although with limited efficacy, particularly in mature bulls [3,17,18]. These antibiotics are also routinely used in semen processing and their use is mandatory for intra-community trade of bovine semen according to the EU Directive 88/407/CEE. However, the prevalence of antimicrobial resistance among *Cfv* isolates has been poorly investigated. Indeed, a genomic island with two genes involved in tetracycline and streptomycin resistance was identified in *Cff* isolates [11,19], but its occurrence in *Cfv* is unknown.

This study aimed to characterize *Cfv* and *Cfv* biovar intermedius (*Cfvi*) isolates, assessing their genomic characteristics, genetic diversity, load of virulence-related genes and in vitro antimicrobial susceptibility.

## 2. Materials and Methods

### 2.1. Campylobacter fetus subsp. venerealis Isolates

The *Cfv* isolates (*n* = 13) were kindly provided by the Starcross Veterinary Investigation Centre from Animal and Plant Health Agency (APHA), United Kingdom, where they were phenotypically identified as *Cfv* (Table 1). The *Cfv* isolates were grown in Columbia agar plates with 5% sheep blood (COS, Biomerieux) for 48 h, under microaerobic conditions (GENbox Microaer, Biomerieux). The subspecies identification (*Cfv*) was further confirmed by the amplification of *nahE* and ISCfe1 sequences, as described by van der Graaf et al. [20].

### 2.2. DNA Isolation

Total DNA was extracted by a rapid boiling method. Briefly, bacterial cells were suspended in 1.5 mL PBS, centrifuged (17,000× *g*, 8 min), the supernatant discarded, and the cellular pellet resuspended in 500 μL of sterile water. After a second centrifugation (17,000× *g*, 5 min), the pellet was resuspended in 100 μL of sterile water and incubated at 95 °C for 15 min. Finally, the lysate was centrifuged (17,000× *g*, 8 min), and the DNA containing supernatant collected. The DNA was quantified using a Nanodrop 2000C spectrophotometer (Thermo Scientific, Waltham, USA) and diluted to 50 ng/μL.

### 2.3. Multilocus Sequence Typing (MLST)

The MLST analysis was performed according to a previously described scheme, based on seven housekeeping genes: *aspA*, *glnA*, *gltA*, *glyA*, *pgm*, *tkt* and *uncA* [5]. The sequence types (STs) were assigned using the Campylobacter MLST database (https://pubmlst.org/campylobacter/ (accessed on 4 September 2020)) sited at the University of Oxford [21]. New alleles and profiles were submitted to this database.

### 2.4. Surface Array Protein and L-Cysteine Transporter Typing

The isolates were classified as *Cfv* or *Cfvi* using a multiplex-PCR for detection of an L-cysteine transporter operon previously described [22]. DNA from *Cfv* strain NCTC 10354 and *Cff* strain NCTC 10842 were used as positive controls.

The surface array protein (sap) serotype (*sapA* and *sapB*) was identified as described before [23], using primers ACF/ACR and BCF/BCR. DNA from *Cfv* strain NCTC 10354 and *Cff* strain NCTC 10842 were used as positive controls in *sapA* and *sapB* PCRs, respectively.

### 2.5. Detection of Putative Virulence Factor Genes Using PCR

The presence of putative VF genes involved in adhesion (*cadF*), invasion (*invA* and *ciaB*) and cytotoxicity (*cdt and pldA*) of host cells [9] was assessed by PCR. For genes *cadF*, *invA*, *ciaB* and *pldA*, primers were designed with Primer-BLAST [24] using gene sequences of *Cfv* NCTC 10354 as template (Table S1). PCR reactions were carried out in 25 µL mixtures, containing 200 μM of each dNTP (4you4 dNTP Mix, Bioron), 400 nM of each primer, 1X reaction buffer (Complete reaction buffer, Bioron), 2 units of DFS-Taq DNA polymerase (Bioron) and 100 ng of DNA. Amplifications were performed in a Doppio thermal cycler (VWR) with the following cycling conditions: initial denaturation step at 94 °C for 2 min, followed by 30 cycles of denaturation at 94 °C for 30 s, annealing temperature for 30 s, extension at 72 °C for 1 min, with a final extension step at 72 °C for 5 min. Detection of *cdtA*, *cdtB* and *cdtC* genes was carried out by PCR as described previously [25].

The presence of genes encoding the most studied T4SS of *Cfv* [10,14], which includes *virB2-virB11*, *virD4* and *fic1* and *fic2* genes were also screened by PCR. Primers were designed with primer-BLAST [24] to target *virB2*, *virB3*-*virB4*, *virB5*, *virB6*, *virB7*, *virB8*, *virB10* and *virD4* genes, using the sequences of *Cfv* NCTC 10354 (GenBank accession no. CP043435.1, loci CFVT_1262–1267, CFVT_1258 and CFVT_1256) as template (Table S1). Genes *fic1*, *fic2*, *virB9* and *virB11* were detected as recently described [26]. PCR mixtures and thermal cycling conditions were performed as described above. The amplification products were separated in a 1.5% agarose gel electrophoresis, stained with ethidium bromide and visualized using a ChemiDoc XRS + System (Bio-Rad).

### 2.6. Detection of parA Gene

The *parA* gene was detected by three PCR assays directed towards distinct nucleotide regions, comprising a conventional PCR with VenSF/VenSR primers [13], two real-time PCR assays, [12] and parA-B assay [26].

### 2.7. Antibiotic Susceptibility Testing

The minimum inhibitory concentrations (MICs) of streptomycin, tetracycline, enrofloxacin and penicillin G were in vitro determined using Etest gradient strips (Biomerieux). *Cfv* colonies grown on COS plates for 48 h were suspended in Brain Heart Infusion (BHI) broth to a turbidity of 1.0 McFarland measured with a Densimat densitometer (Biomerieux). The inoculum was spread on Mueller Hinton agar plates supplemented with 5% horse blood and 20 mg/L of β-NAD (MHF, Biomerieux) and one strip was applied on each agar plate. The concentration gradients of antibiotics in Etest strips used were 0.064–1024 μg/mL for streptomycin, 0.016–256 μg/mL for tetracycline, and 0.002–32 μg/mL for enrofloxacin and penicillin G. Plates were incubated for 48 h at 35 °C in a microaerobic atmosphere, and MICs were read at the point where the zone of inhibition intersected the MIC scale on the Etest strip. *Escherichia coli* ATCC 25922, *Staphylococcus aureus* ATCC 25923, *Enterococcus faecalis* ATCC 29212 and *Pseudomonas aeruginosa* ATCC 27853 were used for quality control, as recommended by the manufacturer.

In the absence of specific interpretative criteria for *Cfv*, the MIC breakpoints of enrofloxacin and tetracycline were defined according to the ciprofloxacin and tetracycline breakpoints defined for *Campylobacter jejuni* and *Campylobacter coli* by the European Commitee on Antimicrobial Susceptibility Testing (EUCAST) [27] (Table 2). Results of penicillin G were interpreted according to the criteria defined for Gram-negative anaerobes by the EUCAST [27] (Table 2). For streptomycin MIC breakpoints, due to the absence of EUCAST breakpoints, results were interpreted according to criteria of The National Antimicrobial Resistance Monitoring System for Enteric Bacteria (NARMS) for *Escherichia coli* [28] (Table 2).

### 2.8. Whole Genome Sequencing of Cfv Strains

Strains IS26-07793, IS16-01257, SA21-221439, SA21-217832 and SA21-217833 were selected for whole genome sequencing (WGS) analysis, as they have unique genomic traits, namely represent a novel ST or harbour the screened T4SS-encoding genes. Strains were grown on blood agar plates supplemented with 5% sheep blood at 37 °C for 48 h and genomic DNA isolated using the DNeasy Blood and Tissue Kit (Qiagen, Hilden, Germany), according to manufacturer’s instructions. Following preparation of DNA libraries, the genomes were sequenced with the Illumina Novaseq Platform at Stabvida (Caparica, Portugal), using 150-bp paired end reads. The reads were de novo assembled in the Pathosystems Resource Integration Center (PATRIC) version 3.6.7 web platform [29], using SPAdes version 3.12.0 [30]. Assembled genomes were submitted to the Comprehensive Genome Analysis service of PATRIC [29], which includes an annotation service using RAST tool kit (RASTtk) [31]. The whole genome shotgun projects of strains IS26-07793, IS16-01257, SA21-221439, SA21-217832 and SA21-217833 were deposited at DDBJ/ENA/GenBank under the accession numbers JAENPS000000000, JAENPT000000000, JAENPU000000000, JAENPV000000000 and JAENPW000000000, respectively.

The genomes were visualized by comparison against reference genomes (*Cfv* strain NCTC 10354 and *Cfv* strain 01/165) using the BLAST Ring Image Generator (BRIG) version 0.95 [32], with an upper identity threshold of 90% and a lower identity threshold of 70%. Genomic islands and T4SS encoding regions VG III [14], PICFV8/T4SS region 1A [9,16], T4SS region 2A and 1F [16] were included in the BRIG analysis.

The MLST allele sequences were extracted from WGS data using the MLST software version 2.0.4 [33] to confirm results of MLST analysis.

### 2.9. Comparative Genomic Analysis

The genomes of the five sequenced strains were compared to 26 whole genome sequences of *Cfv* strains retrieved from the GenBank (Table 3). Genomes were analysed by the Comprehensive Genome analysis service of PATRIC [29], which includes a k-mer-based detection method for antimicrobial resistance genes. Genes assigned to mechanisms of antibiotic inactivation and efflux pumps were considered for this analysis. Additionally, genes *ant(6)-Ib* and *tet*(44) conferring resistance to streptomycin and tetracycline [11] and putative VF encoding genes (*cadF*, *pldA*, *invA*, *ciaB*, *cdtA*, *cdtB* and *cdtC*) were searched by the BLAST tool in the genomes.

The PATRIC’s Family Protein Sorter service [29] was used to evaluate the distribution of protein families across the analysed genomes, and genus-specific families (PLfams) represented in more than 5% and less than 95% of the genomes (2 to 29 genomes), considered accessory protein families, were selected for further analysis. These data were used for the construction of a heat map using Next-Generation Clustered Heat Map (NG-CHM) Builder [34] with hierarchical clustering using the Euclidean distance metric with the complete agglomeration method.

Single nucleotide polymorphisms (SNP) detection and analysis were performed with CSI Phylogeny version 1.4 [35] to reconstruct a phylogenetic tree using the genome of strain NCTC 10354 as reference, with a minimum distance between SNPs set for 10 bp. The tree was illustrated using the Molecular Evolutionary Genetics Analysis (MEGA) X software version 10.1.7 [36] and bootstrap values lower than 70% were hidden.

## 3. Results

### 3.1. Multilocus Sequence Typing of Cfv Isolates

A total of 4 STs were identified among the 13 *Cfv* isolates (Table 4). The allelic profile of isolates IS16-01257 and SA21-221439 was not listed in the PubMLST database and after its submission, the isolates were assigned to ST-71. Two new alleles of *gltA* and *tkt*, assigned respectively to alleles 12 and 14, were deposited in the PubMLST database. The alleles 7 and 12 of *gltA* differ from allele 2, which is the most common, in one nucleotide position. Additionally, alleles 2 and 14 of *tkt* are distinguished by a single nucleotide. Overall, nine isolates were assigned to ST-4 (69.2%), two to ST-71 (15.4%), one to ST-72 (7.7%) and one to ST-73 (7.7%). Interestingly, the isolates from herds I and J were assigned to different STs (herd I—SA21-221825 to ST-4 and SA21-221439 to ST-71; herd J—SA21-217832 to ST-72 and SA21-217833 to ST-73).

### 3.2. Genomic Characterization of Cfv Isolates: Surface Array Protein and L-Cysteine Transporter Typing, Virulence Factor Genes and parA Gene

All the 13 *Cfv* isolates were classified as serotype A (*sapA* positive and *sapB* negative). Most isolates (*n* = 9) revealed a *Cfvi* pattern in L-cysteine transporter PCR (Table 5). The remaining four isolates harbour the L-cysteine transporter encoding operon partially deleted and, consequently, were classified as *Cfv*.

All isolates harbour the CDT operon genes (*cdtABC*), which encode the cytolethal distending toxin, and genes *cadF*, *ciaB*, *invA*, *pldA*, which encode the fibronectin-binding protein, *Campylobacter* invasion antigen B, invasin A and phospholipase A, respectively. These genes were also found in the 26 whole genome sequences of *Cfv* strains, using BLAST search.

Only three isolates (23.1%) were *parA* positive and this result was consistent using the three different assays. Genes *fic1* and *fic2* were found in all *Cfv* isolates, whereas T4SS encoding genes (*virB2*-*virB11* and *virD4*) were present only in *Cfv* isolate IS26-07793 (Table 5). 

### 3.3. Antibiotic Susceptibility Testing of Cfv Isolates

Antibiotic resistances were not found among the *Cfv* isolates. All the 13 isolates were susceptible to tetracycline, streptomycin and enrofloxacin. Eight isolates were categorized as susceptible to penicillin G with standard dose regimen and the remaining 5 isolates (38.5%) were considered susceptible with increased exposure. The MIC values of tetracycline, streptomycin and enrofloxacin for all isolates were below the susceptibility breakpoints. The range of MIC values for each antibiotic and the MIC inhibiting 50% (MIC50) and 90% of the isolates (MIC90) are shown in Table 6.

### 3.4. Whole Genome Sequencing of Five Cfv Strains

The WGS analysis confirmed the PCR result of absence of genes *parA*, *virB2-virB11* and *virD4* of T4SS encoding region 1A, in four of the five sequenced strains. Only *Cfv* strain IS26-07793 harbours the genomic island with the T4SS encoding region 1A [16]. However, all the five strains have other T4SS encoding regions, with gene sequences and gene composition distinct from region 1A (Table 7). 

**Figure 1:**
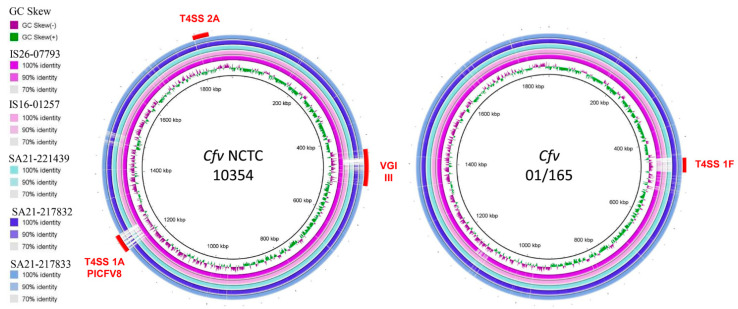
Comparative genomic analysis of *Cfv* strains with reference strains NCTC 10354 and 01/165. Image created using Blast Ring Image Generator version 0.95. The inner ring represents the GC Skew and the remaining rings represent a BLASTN comparison of genomes of IS26-07793, IS16-01257, SA21-221439, SA21-217832 and SA21-217833 with the reference strains NCTC 10354 (**left**) and 01/165 (**right**). Red curved bars indicate chromosomal genomic islands previously identified by other authors (T4SS encoding regions 1A, 1F, 2A) [16].

Plotting the sequenced genomes against the genome of *Cfv* NCTC 10354 as reference showed that all five strains harbour the T4SS encoding region 2A (Figure 1), whereas the region 1A is present only in strain IS16-07793. An additional comparison using the genome of *Cfv* strain 01/165 as reference showed the presence of a T4SS encoding region 1F, in strains SA21-217832 and SA21-217833.

All except strain IS26-07793 harbour a T4SS encoding region 1B, and strains SA21-217832 and SA21-217833 also present T4SS tra/trb encoding regions.

The comparison with the genome of *Cfv* NCTC 10354 revealed the absence of a prophage in VGI III [6] in strains IS16-01257, SA21-221439, SA21-217832 and SA21-217833 within the sap locus.

### 3.5. Comparative Genomic Analysis of Cfv Strains

**Figure 2:**
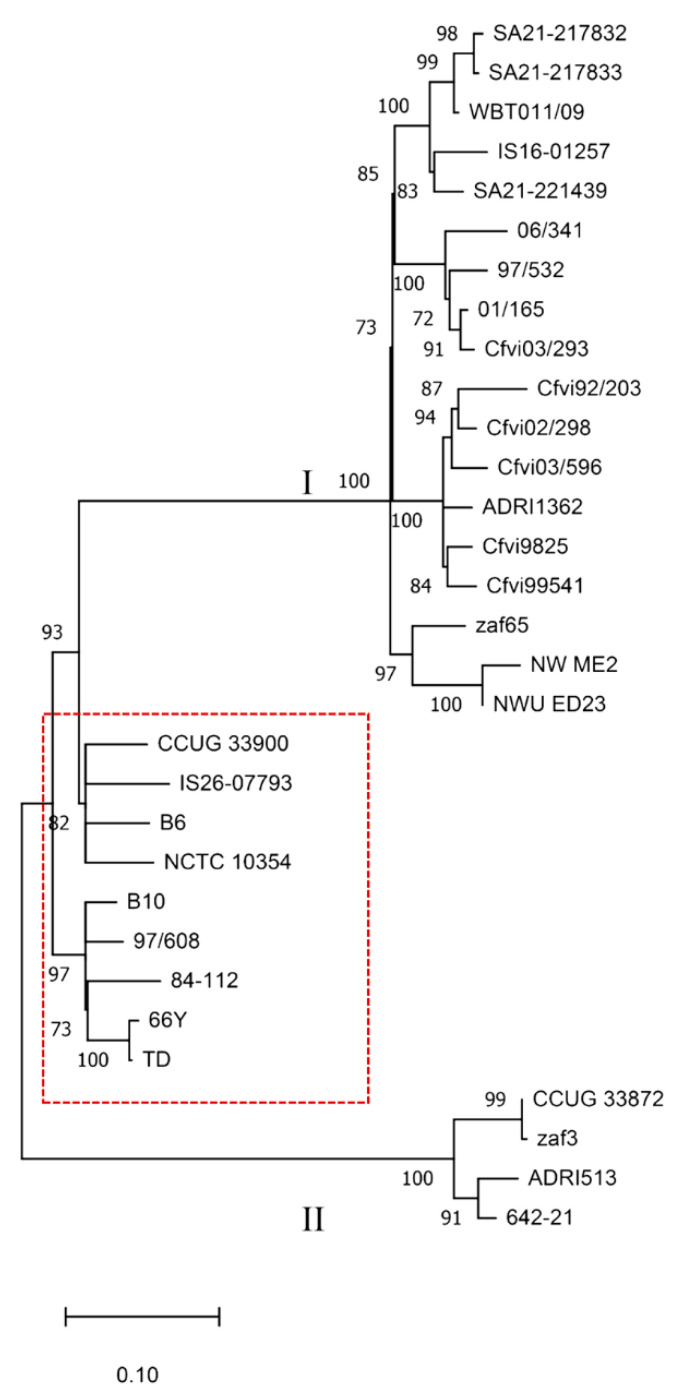
Phylogenetic tree based on single nucleotide polymorphisms (SNP) of 31 *C. fetus* subsp. *venerealis* strains. Numbers at the nodes represent bootstrap values and values lower than 70% were hidden. The red border rectangle separates *Cfv* strains (inside) from strains biotyped as *Cfv* biovar intermedius (outside).

The SNP analysis using the strain NCTC 10354 as reference was based on 1,630,344 nucleotide positions that were common to all genomes. As shown in Figure 2, the strain IS26-07793 is phylogenetically related to strains CCUG 33900, B6 and NCTC 10354. The remaining four sequenced strains are phylogenetically distant from IS26-07793. Strains SA21-217832 and SA21-217833, isolated from the same herd, are highly related despite having different STs. These strains typed as ST-72 and ST-73 are phylogenetically close to IS16-01257 and SA21-221439 typed as ST-71, and the ST-4 WBT011/09.

The phylogenetic tree also shows a clear distinction between strains typed as *Cfvi* or *Cfv* in previous studies [5,22,37]. Although NWU_ED23 and NW_ME2 strains were not typed in these studies, the BLAST search identified the complete L-cysteine transporter encoding gene in NWU_ED23 (contig 72) and the sequence divided into two contigs (contigs 26 and 417) in NW_ME2, which is compatible with a *Cfvi* classification. Overall, nine strains are classified as *Cfv* and 22 as *Cfvi*. *Cfvi* strains are divided into two distant groups (Clusters I and II), with strains CCUG 33872, zaf3, ADRI513 and 642-21 segregated from the remaining *Cfvi* strains. This comparative genomic analysis of *Cfv* strains evidences the presence of similar SNP patterns in isolates from the same geographic region. For instance, strains zaf65, NW_ME2 and NWU_ED23 were isolated from different regions of South Africa. Moreover, *Cfvi* strains sequenced in this study cluster with the strain WBT011/09 from the UK. The results also showed that *Cfvi* strains from Argentina are included in two related clusters grouped with a bootstrap value of 100%.

Regarding the antimicrobial resistance genes, those encoding the multidrug efflux system CmeABC, the broad-specificity multidrug efflux pump YkkCD, the Macrolide-specific efflux protein MacA, the Macrolide export ATP-binding/permease protein MacB and the nitroimidazole resistance protein were found in the genome of the 31 strains under study, whereas the genes *tet*(44) and *ant*(*6*)*-Ib* searched using BLAST were not identified in the genomes under analysis.

**Figure 3:**
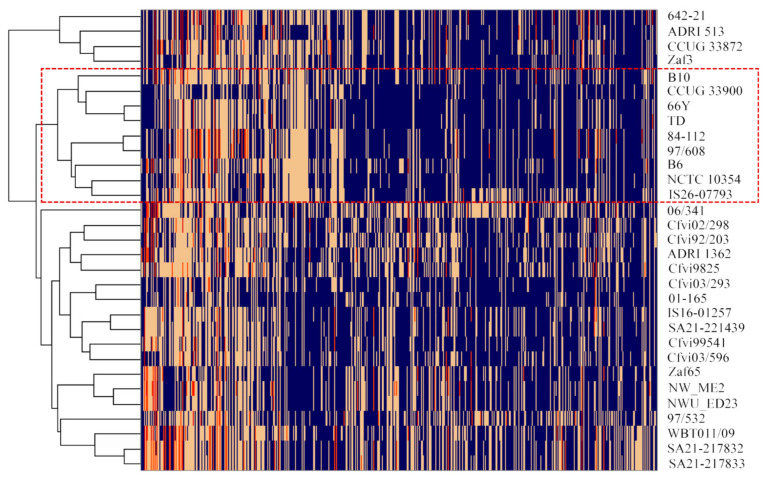
Heat map representing the distribution of accessory protein families (*n* = 540) in the genomes of 31 *Cfv* strains. The absence of the protein family is represented in blue and the number of proteins per family is represented in yellow (*n* = 1), orange (*n* = 2) and red (*n* = 3). *Cfv* strains are grouped by hierarchical clustering, using the Euclidean distance and the complete agglomeration method. The rectangle with red border separates *Cfv* strains (inside) from *Cfv* biovar inter-medius strains (outside).

The analysis of protein families (PLfams) identified 2425 genus-specific protein families, from which 1641 were represented in all the 31 genomes. A total of 1693 proteins are encoded in the genome of 30 or more isolates (≥96.8%), which represent the core gene families considering the commonly accepted cut-off value of 95% and are listed in Supplementary Table S2. The accessory protein families, found in less than 95% of the strains, are represented in a heatmap with hierarchical clustering (Figure 3). A total of 540 accessory protein families were found encoded in the 31 analysed genomes (Table S3), of which 461 have an unknown function (hypothetical proteins). The groups formed based on the accessory protein families almost match those formed based on the SNP phylogenetic tree, with exception of strain *Cfvi* 06/341, 97/532 and UK *Cfvi* strains that were split into two groups. The *Cfvi* strains 642-21, ADRI 513, zaf3 and CCUG33872 are closely related and segregated from the remaining *Cfvi* strains, which is in accordance with SNP analysis. There were no exclusive *Cfv* protein families common to all strains. As expected, two protein families (PLF_194_00014554 and PLF_194_00049089) related to L-cysteine transporters were exclusively found in *Cfvi* strains, with the exception of strain cfvi9925. Excluding this strain, 180 protein families were unique to *Cfvi* and 10 protein families were exclusively represented in the nine *Cfv* genomes.

## 4. Discussion

This study evaluated the virulence potential of *Cfv* strains through genomic and phenotypic approaches and uncovered characteristics of this subspecies that are relevant for BGC control. MLST genotyping showed that most *Cfv* isolates were clustered in ST-4, which is reported to be the most prevalent ST among *Cfv* [5]. Thus, this genotyping method could be considered an effective tool for typing *C. fetus* at the subspecies level [20]. Interestingly, this study revealed a considerable ST diversity and identified three novel STs. These STs differ from ST-4 in one to two nucleotide positions, which denotes the genetic stability of *Cfv*. Nevertheless, the ST variability was higher than expected and the use of MLST for subspecies identification should be further evaluated. In fact, the suitability of MLST for subspecies typing was questionable since the description of one *Cff* strain belongs to ST-4 [38].

The pathogenicity mechanisms of *Cfv* are still unclear. All *Cfv* isolates and 26 genomes from different geographic regions harbour genes encoding the fibronectin-binding protein (*cadF*), Campylobacter invasion antigen B (*ciaB*), invasin A (*invA*), phospholipase A (*pldA*) and cytolethal distending toxin (*cdtABC*), which contribute to the virulence potential of other *Campylobacter* species, playing roles in adhesion, invasion and/or cytotoxicity of host cells [8,39]. The presence of these genes in all genomes of *Cfv* suggests their relevance for host colonization and/or pathogenicity. Further research is needed to understand the contribution of these genes to *Cfv* virulence.

A T4SS encoded by *virB2-virB11* and *virD4* genes, within a genomic island formerly considered unique to *Cfv* [9,10], was suggested as being involved in *Cfv* virulence, namely in cell invasion, cytotoxicity and conjugative DNA transfer [10,14]. Genes encoding this T4SS, corresponding to region 1A [16], were detected in 91% of 67 *Cfv* strains [10]. However, in this study, only 1 out of 13 isolates harbour genes encoding this T4SS, and 7 of the 12 negative strains were isolated from aborted foetuses, which still evidences their pathogenicity even in the absence of this genomic island. The WGS of the five sequenced isolates identified other loci with T4SS encoding genes, with distinct gene sequences from encoding region 1A, whose putative role in *Cfv* pathogenicity or niche specialization have so far not been addressed. These T4SS encoding regions 1B, 1F and 2A lack some *virB/virD4* genes, which require further studies to evaluate their functionality. Other genes that were not analysed in this study may also contribute to the pathogenicity of these isolates and should be evaluated in a larger sample.

Tested by three different PCR assays, only 3 out of 13 isolates harbour the *parA* gene, and this negative status was confirmed in the WGS of four strains. A previous study reported that most *Cfv* isolates from the UK were negative for *parA* gene [40]. The doubt remained whether this resulted from sequence variations in primer-binding sites or from absence of the gene. The present study confirms the absence of the gene in a large proportion of UK strains and clarifies the reason of sensitivity failures of *parA* detection methods for *Cfv* identification [4,20]. In accordance, a recent study [41] reported the absence of this gene in 45% of *C. fetus* genomes proposed as belonging to subspecies *venerealis*, including some of the strains analysed in the present study. The *parA* gene is located in a genomic island, which was already described in *Cff* [11,16] and other *Campylobacter* species [42]. Therefore, the sole use of this molecular marker for *Cfv* identification should be avoided due to its lack of specificity and sensitivity.

To the authors’ best knowledge, this is the first report on antibiotic minimum inhibitory concentrations in *Cfv* field isolates. No antimicrobial resistance to streptomycin, penicillin, tetracycline and enrofloxacin was found in the 13 *Cfv* isolates, and streptomycin and tetracycline resistance genes were not detected in the 31 *Cfv* analysed genomes. These latter genes were identified in *Cff* strains harbouring the *Cfv*-associated genomic island with T4SS encoding genes [11,19]. The antimicrobial susceptibility results of this study are in accordance with a previous study, in which all isolates were susceptible to penicillin, streptomycin and tetracycline and only 5% of the isolates were susceptible to enrofloxacin [43]. Similarly, in another study with *Cfv* isolates from Germany, only 4% of the isolates revealed increased susceptibility to streptomycin and 2% for ciprofloxacin and tetracycline [44]. Antimicrobial resistance data from this study must be regarded with caution, as they refer to a very limited number of isolates from a narrow world geographical region. Nevertheless, they provide proof of concept for the simultaneous presence of in vitro susceptibility to antimicrobials and genes encoding for its resistance. A wide geographical survey with a large sampling is needed to ascertain the presence of antimicrobial resistance in different scenarios.

In contrast, genes encoding two multidrug efflux pumps were detected in all 31 analysed genomes. The CmeABC efflux pump, well-studied in *C. jejuni*, provides resistance to bile salts, heavy metals and antibiotics [45,46]. Mutational analysis of the *cmeB* gene in several *Campylobacter* species, including *C. fetus*, revealed its involvement in antimicrobial resistance [47]. However, results showed that all five sequenced isolates harbour genes encoding this efflux pump and those encoding the ykkCD efflux pump without exhibiting phenotypic resistance to antimicrobials. The role of these efflux pumps in *C. fetus* antimicrobial resistance deserve further research with other antimicrobials, as these systems may act synergistically with other genes conferring antimicrobial resistance.

The resolution provided by MLST to differentiate *Cfv* strains was weak, compared with SNP or accessory protein family analysis. The housekeeping genes used in MLST are very stable among *Cfv* strains, which makes this method very limited for genetic diversity analysis. Although a *Cfv* clonal nature was reported [5], this study identified genomic features that consistently grouped most strains by their SNPs or accessory protein families. Both methods segregated *Cfvis* in two distant clusters, which is indicative of genetic diversity within this biovar. *Cfvi* were also segregated from *Cfv* strains, indicating a higher variability between biovars than the described by the L-cysteine transporter encoding operon. Analysis of protein families revealed 2 proteins (PLF_194_00014554 and PLF_194_00049089) present in *Cfvi* that are absent in *Cfv*. Genes encoding these proteins in *Cfvi* were described as responsible for the phenotypic differences found between biovars [37]. Strain 9825 was the exception, as, while not exhibiting the above two protein families, it was still clustered by SNP and MLST as *Cfvi*. Strain 9825 was initially classified as *Cfvi* in a first study [5], although the isolate failed to produce H_2_S in two other reports [22,37]. This study indicates that this strain is closer to *Cfvi* than to *Cfv*, even lacking genes encoding the L-cysteine transporter.

## 5. Conclusions

This study combined MLST genotyping, VF genes PCR testing, antimicrobial resistance phenotyping, WGS and comparative genomic analysis to evaluate the virulence potential of *Cfv* isolates and strains. Three novel STs were identified by MLST (ST-71, ST-72 and ST-73). Most VF genes common to *Campylobacter* genus were detected, but genes encoding the T4SS, previously regarded as involved in *Cfv* virulence or niche adaptation, were absent in most *Cfv* strains. This indicates that T4SS is not essential for *Cfv* pathogenicity, as strains were isolated from aborted foetuses. The *parA* gene, still used for *Cfv* identification was absent in most *Cfv* strains, which precludes the sole use of this marker for BGC molecular diagnosis. As genes encoding the CmeABC and YkkCD efflux pumps were detected and in vitro antimicrobial resistance towards streptomycin, penicillin, tetracycline and enrofloxacin was not detected, it is demonstrated that the sole presence of those genes is not enough to provide antimicrobial resistance to tested antimicrobials. Genetic diversity was found in isolates from different geographic regions, and WGS and comparative genomic analysis of SNPs and accessory protein families allowed to differentiate biovars *Cfv* from *Cfvi*. Results of this study provided novel knowledge related to *Cfv* virulence potential evaluation and BGC control.

## Figures and Tables

**Table 1:** *Campylobacter fetus* subsp. *venerealis* isolates used in this study.

Isolate	Source of Isolation	Year	Herd
IS26-04236	Aborted fetus	2014	A
IS26-07793	Aborted fetus	2016	B
IS16-01257	Aborted fetus	2013	C
IS14-13272	Aborted fetus	2015	D
IS21-05213	Unknown	2018	E
IS12-08947	Aborted fetus	2019	F
IS21-08727	Aborted fetus	2019	G
IS21-08528	Aborted fetus	2019	H
IS21-08525	Aborted fetus	2019	H
SA21-221439	Bull sheath wash	2019	I
SA21-221825	Bull sheath wash	2019	I
SA21-217832	Bull sheath wash	2019	J
SA21-217833	Bull sheath wash	2019	J

**Table 2:** Minimum inhibitory concentration (MIC) breakpoints for antibiotic susceptibility testing.

Antibiotic	MIC Breakpoints (μg/mL)		Reference
	S≤	R>	
Penicillin G	0.25	0.5	[27]
Enrofloxacin	0.5	0.5	
Tetracycline	2	2	
Streptomycin	16	32	[28]

MIC—minimum inhibitory concentration; S—susceptible, standard dose regimen; R—resistant.

**Table 3:** Genomes of *Campylobacter fetus* subsp. *venerealis* strains used for the comparative genomic analysis.

*Cfv* Strain	GenBank Accession	Country	Source of Isolation (Bovine)	Assembly Level
NCTC 10354	CP043435.1	United Kingdom	Vagina	complete
WBT011/09	LMBI00000000.1	United Kingdom	Unknown	contig
CCUG 33900	LREV00000000.1	France	Aborted fetus	contig
CCUG 33972	LREU00000000.1	Czech Republic	Unknown	contig
cfvi03/293	CP006999.2	Argentina	Aborted fetus	complete
cfvi9825	LRES00000000.1	Argentina	Aborted fetus	contig
cfvi97/532	LRER00000000.1	Argentina	Vagina	contig
cfvi92/203	LRVL00000000.1	Argentina	Vagina	contig
cfvi03/596	LRAM00000000.1	Argentina	Aborted fetus	contig
cfvi02/298	LRVK00000000.1	Argentina	Aborted fetus	contig
01/165	CP014568.1	Argentina	Mucus	complete
97/608	CP008810.1	Argentina	Unknown	complete
99541	ASTK00000000.1	Argentina	Preputial sample	contig
ADRI1362	LREX00000000.1	Argentina	Unknown	contig
06/341	SOYW00000000.1	Argentina	Aborted fetus	contig
cfvB10	LRET00000000.1	USA	Unknown	contig
84-112	HG004426.1	USA	Unknown	complete
TD	JPPC00000000.1	Canada	Preputial sample	contig
66Y	JPQC00000000.1	Canada	Preputial sample	contig
B6	AJMC00000000.1	Australia	Vagina	scaffold
642-21	AJSG00000000.1	Australia	Uterus	scaffold
ADRI513	LRFA00000000.1	Australia	Unknown	contig
zaf3	LREZ00000000.1	South Africa	Aborted fetus	contig
zaf65	LREY00000000.1	South Africa	Unknown	contig
NW_ME2	JAATTN000000000.1	South Africa	Unknown	contig

**Table 4:** Sequence types and corresponding allelic profiles.

ST	Isolates	Alleles						
		*aspA*	*glnA*	*gltA*	*glyA*	*pgm*	*tkt*	*uncA*
4	IS26-04236 IS26-07793 IS14-13272 IS21-0513 IS21-08528 IS12-08947 IS21-08525 SA21-221825 IS21-08727	1	2	2	2	1	2	1
71	IS16-01257 SA21-221439	1	2	7	2	1	2	1
72	SA21-217832	1	2	12	2	1	14	1
73	SA21-217833	1	2	2	2	1	14	1

**Table 5:** Genomic characteristics of the *Cfv* isolates.

Isolate	Sap Type	L-Cysteine Transporter Profile	*parA* Gene	*fic* Genes	T4SS Enconding Genes
IS26-04236	A	*Cfv*	+	+	−
IS26-07793	A	*Cfv*	+	+	+
IS16-01257	A	*Cfvi*	−	*+*	−
IS14-13272	A	*Cfv*	+	*+*	−
IS21-05213	A	*Cfv*	−	*+*	−
IS21-08528	A	*Cfvi*	−	*+*	−
IS12-08947	A	*Cfvi*	−	*+*	−
IS21-08525	A	*Cfvi*	−	*+*	−
SA21-221439	A	*Cfvi*	−	*+*	−
SA21-221825	A	*Cfvi*	−	*+*	−
IS21-08727	A	*Cfvi*	−	+	−
SA21-217832	A	*Cfvi*	−	*+*	−
SA21-217833	A	*Cfvi*	−	*+*	−

+ amplification of the target region; − absence of amplification.

**Table 6:** Minimum inhibitory concentrations of selected antibiotics for *Cfv* isolates.

Antibiotic	MIC (µg/mL)		
	Range	50%	90%
Tetracycline	0.047–0.064	0.064	0.064
Streptomycin	0.5–3.0	2.0	2.0
Enrofloxacin	0.032–0.125	0.064	0.094
Penicillin	0.047–0.38	0.25	0.38

**Table 7:** T4SS encoding genes found in different genomic regions.

Region	T4SS Encoding Genes											
	*virD4*	*virB11*	*virB10*	*virB9*	*virB8*	*virB7*	*virB6*	*virB5*	*virB4*	*virB3*	*virB2*	*virB1*
1A												
1B												
1F												
2A												

Blue or white colored cells represent presence or absence of the gene, respectively. T4SS encoding regions based on a previous classification [16].

## Data Availability

The whole genome shotgun projects of strains IS26-07973, IS16-01257, SA21-221439, SA21-217832 and SA21-217833 were deposited at DDBJ/ENA/GenBank under the accession numbers JAENPS000000000, JAENPT000000000, JAENPU000000000, JAENPV000000000 and JAENPW000000000, respectively. Additional data presented in this study are available in Supplementary Tables S1–S3.

## Supplementary Materials

The following are available online at https://www.mdpi.com/2076-2607/9/2/340/s1, Table S1: Primers designed in this study for detection of putative virulence genes and T4SS-encoding genes, Table S2: Genus-specific protein families (PLFams) represented in more than 95% of the *Cfv* genomes; Table S3: Genus-specific protein families (PLFams) represented in more than 5% and less than 95% of the *Cfv* genomes.
