# Supplementary material for: Genomic and Phenotypic Characterization of Campylobacter fetus subsp. venerealis Strains
Source: Microorganisms. 2021 Feb 9;9(2):340. doi: 10.3390/microorganisms9020340 (PMC7916060; doi:10.3390/microorganisms9020340)
Supplement: Supplementary file 1 [file microorganisms-09-00340-s001.zip › Table S1.pdf]

Table S1. Primers designed in this study for detection of putative virulence genes and T4SS-encoding genes

Target	Primers	Annealing temperature	Product size (bp)
cadF	Fw: ATGAGCCGTTTAGCGTGGTT	60 ºC	234
	Rv: GAGTTGATGATTGCGCTTTGGA		
ciaB	Fw: TTCACGCTGCGATAGGGTTT	56 ºC	337
	Rv: GAGCGCTACTGCGTGAGTAT		
invA	Fw: GCGTTTGACCATCGTATGGAG	58 ºC	243
	Rv: CTCCAACTTATCCGCTTGAGTG		
pldA	Fw: CGCATAGCATTTGGCAGCAA		300
	Rv: ACTCTTTTGCTATCTGCCACCA		
virB2	Fw: TCTTTTGTTCGCTGCTGGTG		153
	Rv: CTTACTGTTTGACCGCCCCA		
virB3-virB4	Fw: ACTTATGGCGGCAGAGGATG		482
	Rv: CGCCACTTTGACCAAGAACG		
virB5	FW: GCATTCCAGTTGTAGATGGTGC		358
	Rv: GAGCATTGATCTTTTCCGCC		
virB6	Fw: TTCCAACAGCCATACCGCAT	60 ºC	426
	Rv: TTTGGGCTGGACTTATGGGC		
virB7	Fw: CTTGTTGGTTGCACAAGCGT	58 ºC	256
	Rv: GCGCTATCTCCTGATTGCCA		
virB8	Fw: AACCAGCGTAAATGAAGCCG		385
	Rv: TGTCGTAAGCGAACTAGGTTGA		
virB10	Fw: TGATTTCGCTCCACACGACA		741
	Rv: TTGCCTACGAGCAACCAGAG		
virD4	Fw: AATGGCAAAATTCCAAGTCG	56 ºC	286
	Rv: CCTTGCCTGATTGTTCGATT		
